# Total coronary atherosclerotic plaque burden is associated with myocardial ischemia in non-obstructive coronary artery disease

**DOI:** 10.1016/j.ijcha.2021.100831

**Published:** 2021-06-30

**Authors:** Ingeborg Eskerud, Eva Gerdts, Terje H. Larsen, Judit Simon, Pál Maurovich-Horvat, Mai Tone Lønnebakken

**Affiliations:** a Department of Clinical Science, University of Bergen, PO box 7804, N-5020 Bergen, Norway; b Department of Heart Disease, Haukeland University Hospital, PO box 1400, N-5021 Bergen, Norway; c Department of Biomedicine, University of Bergen, Postbox 7804, N-5020 Bergen, Norway; d MTA-SE Cardiovascular Imaging Research Group, Heart and Vascular Center, Semmelweis University, 68 Városmajor Street, Budapest, Hungary; e Medical Imaging Centre, Semmelweis University, 18 Hataror ut, 1122, Budapest, Hungary

**Keywords:** Non-obstructive coronary artery disease, INOCA, Myocardial ischemia, Coronary computed tomography angiography, Coronary plaque burden, Coronary plaque volume

## Abstract

**Aim:**

Whether the total coronary atherosclerotic plaque burden is independently associated with myocardial ischemia in non-obstructive coronary artery disease (CAD) is not well established. We aimed to test the association of total plaque burden quantified by coronary computed tomography angiography (CCTA) with myocardial ischemia in patients with chronic coronary syndrome and non-obstructive CAD.

**Methods:**

We included 125 patients (age 62 ± 9 years, 58% women) with chronic coronary syndrome and non-obstructive CAD (stenosis < 50%) by CCTA, who were grouped according to presence or absence of myocardial ischemia by myocardial contrast stress echocardiography. Total plaque burden was quantified by CCTA as the total plaque volume in the main coronary arteries, and positive remodelling was defined as remodelling index > 1.10.

**Results:**

Patients with myocardial ischemia (n = 66) had higher total plaque burden (847 ± 245 mm^3^ vs. 758 ± 251 mm^3^, p = 0.049) and higher left ventricular (LV) mass index (42.1 ± 9.9 g/m^2.7^ vs. 37.3 ± 8.0 g/m^2.7^, p = 0.004), while age, sex, prevalence of hypertension, diabetes, calcium score and positive remodelling did not differ between the groups (all p > 0.05). In multivariable regression analysis, total plaque burden remained associated with presence of myocardial ischemia (OR 1.02, 95% CI 1.00–1.04, p = 0.045) independent of age, sex, hypertension, diabetes, LV mass index, coronary calcium score and positive remodelling.

**Conclusion:**

Total coronary artery plaque burden by CCTA was independently associated with myocardial ischemia in patients with non-obstructive CAD. Whether plaque quantification is useful for clinical management of patients with non-obstructive CAD should be tested in prospective studies.

ClinicalTrials.gov: Identifier NCT01853527.

## 1 Introduction

In chronic coronary syndrome, coronary computed tomography angiography (CCTA) is now recommended as an initial diagnostic test for patients with low to moderate pre-test probability of coronary artery disease (CAD) [1]. As non-obstructive CAD (stenosis < 50%) is detected in 30% of patients with chronic coronary syndrome examined with CCTA, the number of patients with non-obstructive CAD is expected to increase in the years to come [2]. In contrast to earlier conceptions that non-obstructive CAD was a harmless condition, it has been shown that patients with non-obstructive CAD may have myocardial ischemia (INOCA) [3], increased risk of myocardial infarction [4] and death [2], [5]. Moreover, improved cardiovascular prevention therapy after CCTA, including statin treatment, seems to reduce the cardiovascular risk also in non-obstructive CAD [6], [7]. However, there is a need to improve CCTA phenotyping of patients with non-obstructive CAD in order to obtain evidence-based therapy [3].

The total burden of coronary atherosclerosis can be evaluated non-invasively by computed tomography (CT). Coronary calcium score is in clinical use as a marker of the total burden of atherosclerosis [1], and higher calcium score has been associated with presence of myocardial ischemia in chronic coronary syndrome [8]. However, calcium score does not reflect non-calcified plaque, which has been associated with ischemia in non-obstructive CAD [9]. In contrast to calcium scoring, CCTA provides quantification of both calcified and non-calcified atherosclerotic plaque, thus providing a more accurate estimate of the total atherosclerotic plaque burden [10]. Moreover, a recent analysis from the CORE320 (Coronary Artery Evaluation using 320-row Multidetector Computed Tomography Angiography and Myocardial Perfusion) study demonstrated that patients with non-obstructive CAD and myocardial ischemia have a larger coronary artery plaque burden by CCTA than patients with non-obstructive CAD and normal myocardial perfusion [11]. However, the independency of other established cardiovascular risk factors has not been explored. Thus, the aim of our study was to evaluate whether total coronary artery plaque burden by CCTA was associated with myocardial ischemia in patients with non-obstructive CAD and chronic coronary syndrome, independent of cardiovascular risk factors.

## 2 Methods

### 2.1 Patient population

The Myocardial Ischemia in Non-obstructive Coronary Artery Disease (MicroCAD) study is a cross-sectional study including participants with chronic coronary syndrome and non-obstructive CAD by CCTA. Inclusion and exclusion criteria have been previously described in detail [12], [13]. In brief, the participants were prospectively included from patients with symptomatic chronic coronary syndrome clinically diagnosed with non-obstructive CAD by CCTA at the Department of Heart Disease, Haukeland University Hospital, Bergen, Norway from May 2013 until November 2014. Inclusion criteria were exercise-induced angina pectoris and/or dyspnea for at least 6 months, non-obstructive CAD (presence of ≥ one stenosis with lumen diameter reduction 1–49% in any coronary artery) by clinically indicated CCTA, age > 30 years and presence of at least one cardiovascular risk factor. All participants signed informed consent. The MicroCAD project was approved by the regional ethical committee, performed according to the 1975 Declaration of Helsinki, and is registered at ClinicalTrials.gov with identifier NCT01853527.

**Fig. 1:**
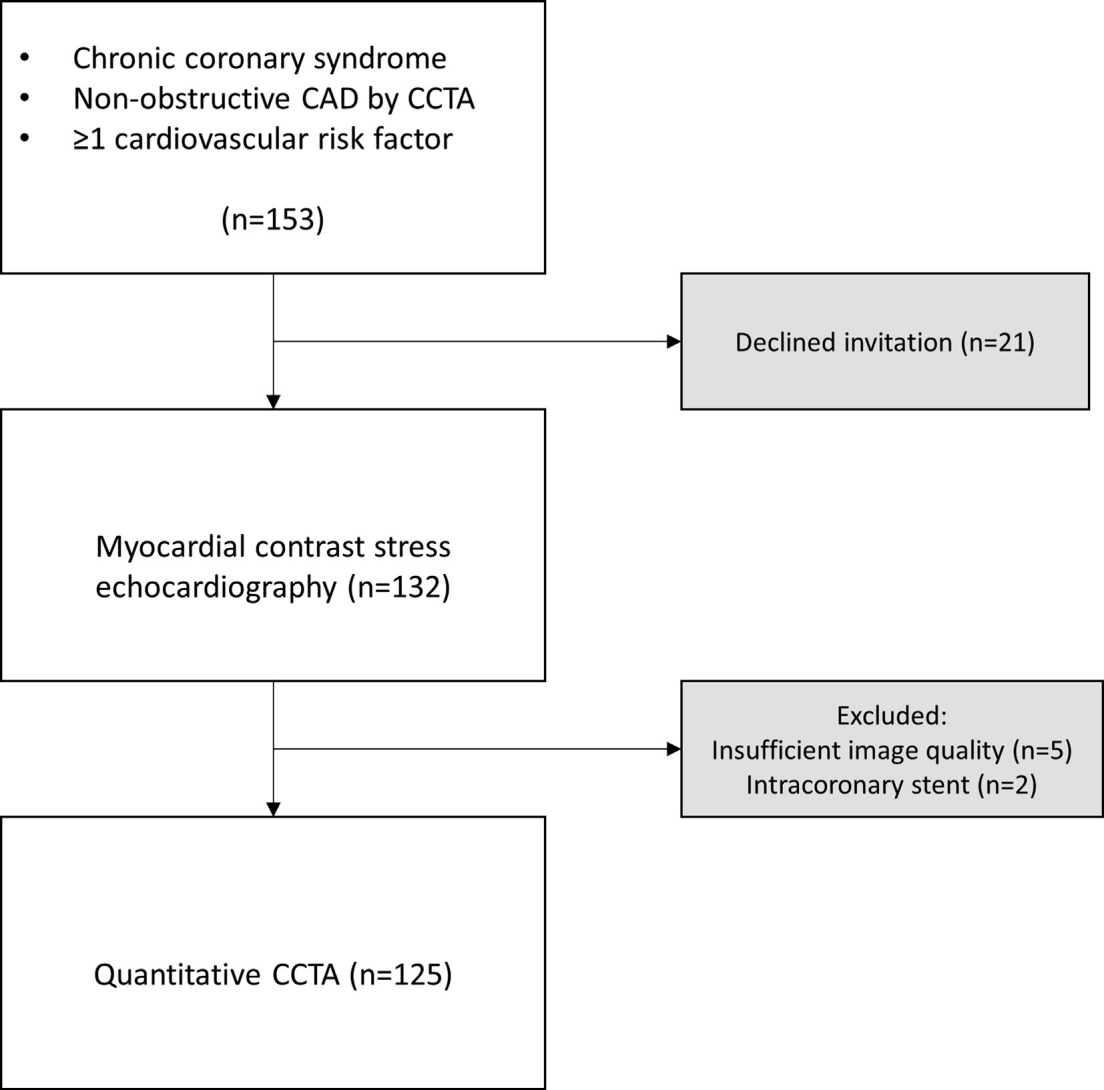
Flow chart of study participants included in the analysis. CAD, coronary artery disease; CCTA, coronary computed tomography angiography.

For the current analysis, we excluded two patients because of intracoronary stents and five patients because of insufficient image quality in CCTA for quantitative analysis, leaving 125 patients eligible (Fig. 1).

### 2.2 Cardiovascular risk factors

The patients reported cardiovascular risk factors, medical history and use of medication on a standardized questionnaire before undergoing CCTA [12]. Family history of premature CAD was considered present if occurring in a first-degree relative under the age of 65 in women and under the age of 55 in men. Obesity was defined as body mass index (BMI) ≥ 30 kg/m^2^. Hypertension was defined as history of hypertension, use of antihypertensive drugs or office systolic blood pressure ≥ 140 mmHg and/or diastolic blood pressure ≥ 90 mmHg at the clinical visit [14]. Fasting blood samples were collected for analyses of serum lipid profile, hemoglobin A1_c_ (HbA1c) and creatinine. Glomerular filtration rate was estimated using the Chronic Kidney Disease Epidemiology Collaboration (CKD-EPI) formula [15]. Diabetes was defined as history of diabetes or HbA1c ≥ 48 mmol/mol [16].

### 2.3 Coronary computed tomography angiography acquisition

CT images were obtained by a dual-source CT scanner with 2 × 128 slices (Somatom Definition Flash, Siemens, Germany) with electrocardiographic (ECG) -gated acquisitions in accordance with current guidelines, as previously described [12], [17]. Experienced readers analyzed the images for detection of coronary artery stenosis using a modified 20-segment American Heart Association model [18], [19]. Calcium score was estimated by the Agatston method [19]. Non-obstructive CAD was defined as presence of ≥ one stenosis with lumen diameter reduction 1–49% in any coronary artery segment.

### 2.4 Quantitative coronary computed tomography angiography image analysis

**Fig. 2:**
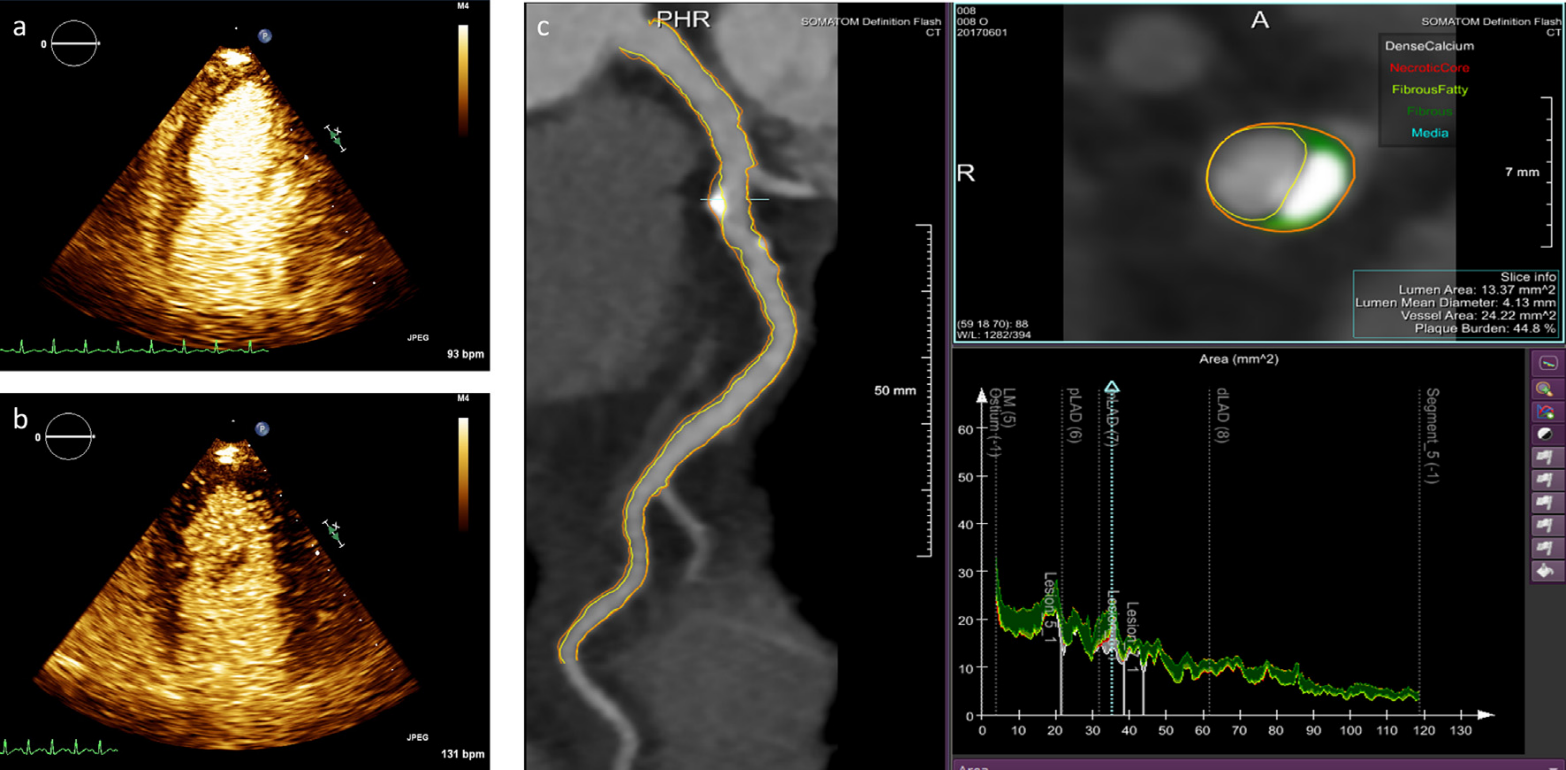
Stress induced myocardial ischemia and quantification of coronary artery plaque burden in a patient with non-obstructive coronary artery disease and chronic coronary syndrome. Myocardial contrast dobutamine stress echocardiography demonstrated normal perfusion at rest (panel a) and myocardial ischemia with delayed myocardial contrast enhancement in the apical part of the left ventricle at peak dobutamine stress (panel b). Quantification of coronary artery plaque burden by coronary computed tomography angiography is shown in panel c.

Quantitative plaque assessment was performed on anonymized CCTA images by a single reader (IE) blinded to clinical and echocardiographic data. Quantitative CCTA was performed using a validated software tool (QAngio CT Research Edition version 3.1.4.2, Medis medical imaging systems, Leiden, The Netherlands) on an offline workstation (Fig. 2) [20], [21]. The coronary artery segments were assessed following the 17-segment model of the Society of Cardiovascular Computed Tomography [19]. Measurements were performed in the left main stem, the left anterior descending artery, the left circumflex artery, and the right coronary artery with lumen diameter > 2.0 mm. The outer and inner walls of the coronary arteries were detected automatically and adjusted manually when needed. The total plaque burden was defined as the total plaque volume, which was calculated by subtracting the lumen volume from the coronary artery vessel wall volume in each patient. Plaque composition was automatically determined as low-attenuation, fibrous-fatty, fibrous, or dense calcium according to the radio density in Hounsfield Units (HU), with thresholds adjusted to the lumen contrast density [22]. Non-calcified plaque volume was taken as the sum of low-attenuation, fibrous-fatty, and fibrous plaque volumes. Segment involvement score was calculated as the total number of coronary artery segments with plaque (range 0–17). Remodelling index was defined as the luminal diameter at maximum stenosis divided by the luminal diameter in a proximal healthy segment. Positive remodelling was taken as remodelling index > 1.10. Intraobserver reliability of total coronary artery plaque volume measurements was estimated from CCTA images of 10 randomly selected patients analyzed twice by the same reader (IE). Interobserver reliability was determined by assessing total coronary artery plaque volume by two independent readers (IE and JS) in 20 randomly selected patients.

### 2.5 Conventional echocardiography

Echocardiography was performed in all patients following a standardized protocol, as previously described [12], [13]. Quantitative echocardiography was performed in line with the current joint guidelines of the American Society of Echocardiography and European Association of Cardiovascular Imaging [23]. All echocardiographic images were analyzed blinded to clinical and CCTA data, and proofread by the same experienced reader (MTL). Left ventricular ejection fraction was assessed by Simpson’s biplane method. Left ventricular mass was calculated using a prognostically validated equation and indexed for height in meters^2.7^ [24]. Relative wall thickness was calculated as posterior wall thickness/left ventricular internal radius ratio [23].

### 2.6 Myocardial contrast stress echocardiography and myocardial ischemia

Myocardial contrast dobutamine stress echocardiography with real-time low-mechanical index imaging and destruction replenishment was performed following current guidelines [25]. Ultrasound contrast agent (SonoVue, Bracco, Milan, Italy) was given intravenously as 1 ml bolus followed by 1 ml/hour infusion with a rotating infusion pump (VueJet, Bracco, Milan, Italy). Myocardial perfusion was scored using apical 2-, 3- and 4-chamber views at rest and peak dobutamine stress, defined as 85% of maximum age-predicted (200 – age) heart rate during stress echocardiography [25]. Myocardial perfusion was scored visually as normal or delayed in each segment of the left ventricle, using a 17- segment model [25]. Myocardial ischemia was defined as delayed contrast replenishment two heartbeats after flash at peak stress in any left ventricular segment (Fig. 2) [25]. The ischemic burden was defined as the number of ischemic left ventricular segments. We have previously demonstrated good intrareader reliability by myocardial contrast stress echocardiography [26].

### 2.7 Statistical analysis

Data analysis was performed using IBM SPSS Statistics version 25 (IBM Corporation, Armonk, NY, USA) and GraphPad Prism version 8.0.1 (GraphPad Software, San Diego, California, USA). Intra- and interobserver reliability were reported as intraclass correlation coefficient (ICC) with 95% confidence interval. The patients were grouped according to presence or absence of myocardial ischemia by myocardial contrast stress echocardiography. The groups were compared by unpaired Student‘s *t*-test for continuous variables with normal distribution, independent samples Mann-Whitney *U* test for continuous variables with skewed distribution (calcium score and calcified plaque), and chi-square test for categorical variables. The results are presented as mean ± standard deviation or median with interquartile range for continuous variables and percentages for categorical variables. To further explore the association between plaque burden, plaque composition, and myocardial ischemia, the patients were grouped according to quartiles of total plaque volume, non-calcified plaque volume, calcified plaque volume, and calcium score. We then compared the frequency of myocardial ischemia across the quartiles with chi-square test of trend. Uni- and multivariable logistic regression analyses were used to find covariables associated with presence of myocardial ischemia, and the results are reported as odds ratio (OR) and 95% confidence interval. Univariable and multivariable linear regression analyses were performed to find covariables associated with the ischemic burden, with reporting of standardized beta coefficient (β). Variables were entered into the models based on clinical judgment and results from univariable analyses. Total plaque volume and non-calcified plaque volume were analyzed in separate multivariable models in order to avoid overfitting. Level of significance was two-sided p < 0.05 in all analyses.

## 3 Results

### 3.1 Clinical characteristics

In the total study population, the mean age was 62 ± 9 years, and 72 (58%) were women (Table 1). Myocardial ischemia was found in 66 patients (Table 1). None of the patients had prior coronary artery bypass grafting, and one patient reported prior myocardial infarction. Groups with and without myocardial ischemia did not differ with respect to age, sex, prevalence of hypertension, diabetes, or blood pressure (Table 1). However, use of acetylsalicylic acid was more common in patients with myocardial ischemia, while use of statin was similar in the two groups (Table 1). By myocardial contrast stress echocardiography, 111 (89%) reached age-predicted heart rate at peak dobutamine stress. Eight of the 13 subjects with lower than predicted heart rate at peak stress had myocardial ischemia. In subjects with ischemia, the mean extent of myocardial ischemia was 5.2 ± 2.5 left ventricular segments.

**Table 1:** Clinical characteristics of the total study population and in patients with and without myocardial ischemia.

	Total population (n = 125)	Ischemia (n = 66)	No ischemia (n = 59)	p
*Clinical characteristics*				
Age (years)	62 ± 9	63 ± 9	62 ± 9	0.355
Female sex	72 (58%)	36 (55%)	36 (61%)	0.465
BMI (kg/m^2^)	27.6 ± 4.5	27.1 ± 4.1	28.1 ± 4.9	0.247
Obesity	28 (22%)	10 (15%)	18 (31%)	0.040
Hypertension	92 (74%)	53 (80%)	39 (67%)	0.097
Diabetes	17 (14%)	8 (13%)	9 (16%)	0.862
Current smoker	18 (14%)	8 (12%)	10 (17%)	0.445
Family history of premature CAD	71 (57%)	34 (52%)	37 (63%)	0.139
Chest pain ≥ 6 months	94 (75%)	50 (76%)	44 (75%)	0.879
Dyspnea ≥ 6 months	83 (66%)	44 (67%)	39 (66%)	0.947
Systolic blood pressure (mmHg)	134 ± 16	134 ± 16	135 ± 16	0.774
Diastolic blood pressure (mmHg)	78 ± 13	78 ± 12	78 ± 13	0.927
Heart rate (bpm)	65 ± 12	66 ± 11	63 ± 12	0.167
HbA_1c_ (mmol/mol)	39 ± 9	39 ± 7	39 ± 10	0.986
Estimated GFR (mL/min/1.73 m^2^)	86 ± 14	87 ± 15	84 ± 13	0.292
Total serum cholesterol (mmol/L)	5.1 ± 1.3	5.1 ± 1.4	5.0 ± 1.1	0.780
Serum HDL cholesterol (mmol/L)	1.5 ± 0.4	1.5 ± 0.5	1.5 ± 0.4	0.850
Serum LDL cholesterol (mmol/L)	3.3 ± 1.2	3.3 ± 1.3	3.2 ± 1.0	0.791
Serum triglycerides (mmol/L)	1.48 ± 0.98	1.56 ± 0.85	1.40 ± 1.11	0.327

*Medical therapy*				
Acetylsalicylic acid	51 (41%)	32 (49%)	19 (32%)	0.027
Statin	46 (37%)	25 (38%)	21 (36%)	0.543
Antihypertensive treatment	64 (51%)	34 (52%)	30 (51%)	0.663
Beta blocker	34 (27%)	14 (21%)	20 (34%)	0.154
Calcium channel blocker	21 (17%)	15 (23%)	6 (10%)	0.056
Angiotensin converting enzyme inhibitor	8 (6%)	4 (6%)	4 (7%)	0.936
Angiotensin 2 receptor inhibitor	38 (30%)	25 (38%)	13 (22%)	0.039

BMI, body mass index; CAD, coronary artery disease; bpm, beats per minute; HbA1c, hemoglobin A1c, GFR, glomerular filtration rate; HDL, high-density lipoprotein; LDL, low-density lipoprotein.

### 3.2 Coronary artery plaque characteristics and myocardial ischemia

**Fig. 3:**
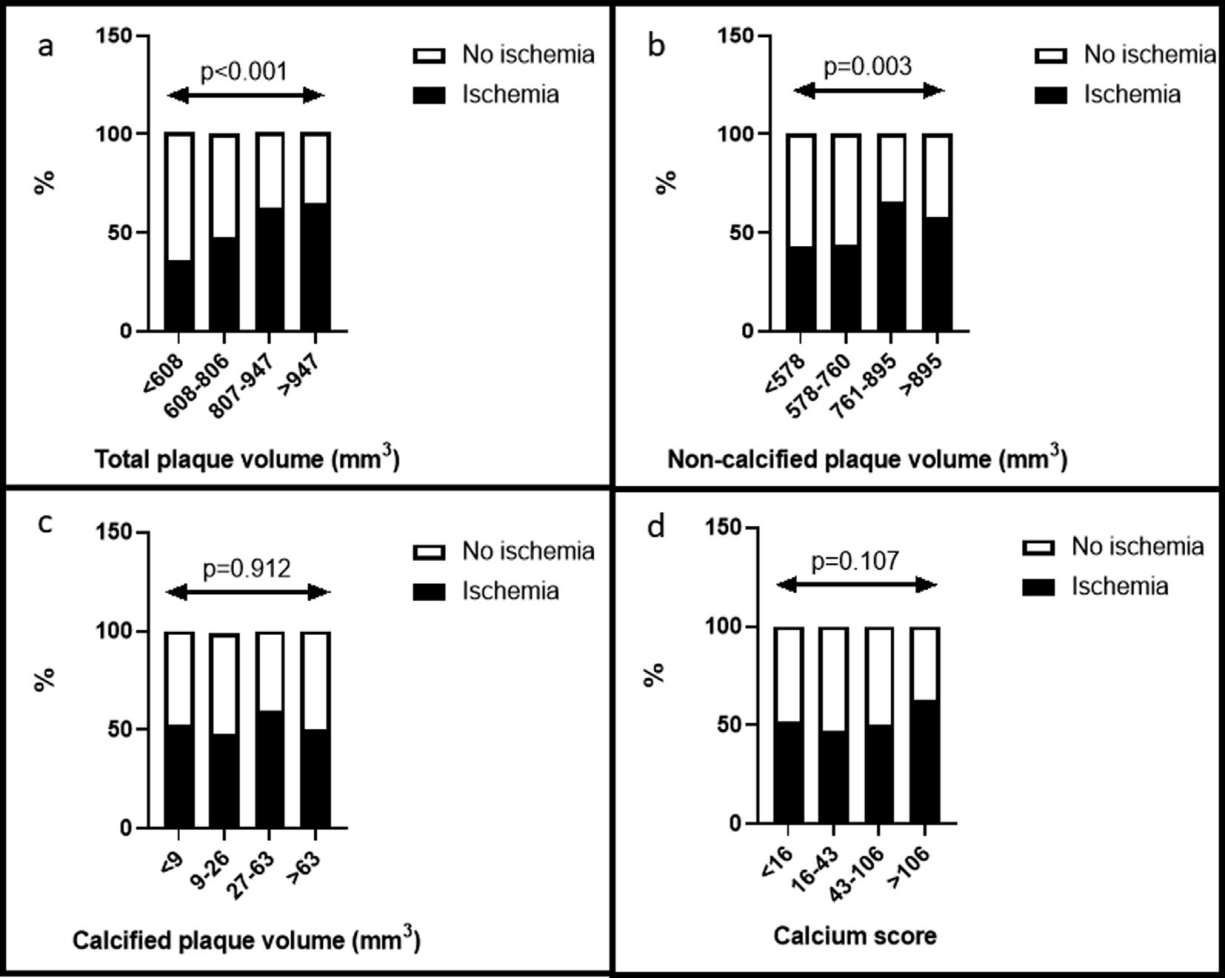
Prevalence of myocardial ischemia by quartiles of different measures of plaque burden. Bar graphs illustrating prevalence of myocardial ischemia by quartiles of total plaque volume (panel a), non-calcified plaque volume (panel b), calcified plaque volume (panel c) and calcium score (panel d). p-values are reported for trends.

Intra- and interobserver reliability of total coronary artery plaque volume was excellent with ICC 0.96 (95% CI = 0.86–0.99) and ICC 0.91 (95% CI = 0.78–0.97), respectively. Time from CCTA to myocardial contrast stress echocardiography was 133 days (interquartile range 97, 187). The total plaque volume and non-calcified plaque volume were both higher in patients with myocardial ischemia, while calcified plaque volume did not differ between the groups (Table 2). In addition, calcium score, segment involvement score and prevalence of positive remodelling were also comparable between groups (Table 2). When grouping patients according to quartiles of quantifiable plaque volumes and calcium score, the prevalence of myocardial ischemia increased with increasing total plaque volume and non-calcified plaque volume (Fig. 3, panel a and b). The prevalence of ischemia did not differ between quartile groups of calcified plaque volume or calcium score (Fig. 3, panel c and d).

**Table 2:** Coronary computed tomography angiography and echocardiographic characteristics in the total study population and in patients with and without myocardial ischemia.

	Total (n = 125)	Ischemia (n = 66)	No ischemia (n = 59)	p
*Coronary computed tomography angiography*				
Total plaque volume (mm^3^)	805 ± 251	847 ± 245	758 ± 251	0.049
Total non-calcified plaque volume (mm^3^)	749 ± 233	788 ± 228	706 ± 233	0.049
Total low-attenuation plaque volume (mm^3^)*	10 (6, 17)	10 (7, 15)	11 (6, 20)	0.205
Total calcified plaque volume (mm^3^)*	27 (9, 63)	28 (10, 57)	23 (9, 66)	0.739
Total vessel volume (mm^3^)	2609 ± 849	2644 ± 762	2570 ± 942	0.630
Total lumen volume (mm^3^)	1804 ± 704	1797 ± 619	1812 ± 794	0.907
Total coronary artery length (mm)	250 ± 59	253 ± 57	246 ± 62	0.544
Calcium score*	43 (16, 106)	46 (16, 125)	38 (13, 83)	0.333
Segment involvement score	2.6 ± 1.6	2.8 ± 1.8	2.4 ± 1.3	0.178
Remodelling index	1.06 ± 0.14	1.07 ± 0.14	1.06 ± 0.15	0.702
Positive remodelling	45 (36%)	26 (39%)	19 (32%)	0.403

*Localization of non-obstructive coronary artery disease*				
Left main stem	14 (11%)	10 (15%)	4 (7%)	0.140
Left anterior descending artery	108 (86%)	58 (88%)	50 (85%)	0.606
Left circumflex artery	42 (34%)	29 (44%)	13 (22%)	0.009
Right coronary artery	47 (38%)	25 (38%)	22 (37%)	0.958

*Echocardiography*				
Left ventricular internal diastolic dimension (mm)	45 ± 5	45 ± 6	45 ± 5	0.933
Left ventricular internal systolic dimension (mm)	29 ± 5	29 ± 5	29 ± 5	0.959
Septal thickness (mm)	12 ± 2	12 ± 2	11 ± 2	0.005
Posterior wall thickness (mm)	9 ± 2	9 ± 2	9 ± 2	0.319
Left ventricular ejection fraction (%)	62 ± 7	63 ± 7	60 ± 7	0.042
Left ventricular mass index (g/m^2.7^)	39.8 ± 9.4	42.1 ± 9.9	37.3 ± 8.0	0.004
Relative wall thickness	0.42 ± 0.11	0.43 ± 0.12	0.41 ± 0.10	0.471

*Median and interquartile range.

### 3.3 Determinants of myocardial ischemia

Determinants of myocardial ischemia are reported in Table 3. In multivariable logistic regression analysis, total plaque volume remained independently associated with presence of myocardial ischemia after adjusting for cardiovascular risk factors, calcium score and positive remodelling (Table 3, Model 1), while the association of non-calcified plaque volume with presence of myocardial ischemia was attenuated after multivariable adjustment (Table 3, Model 2). Determinants of the ischemic burden are shown in Table 4. Larger total plaque volume was independently associated with increasing ischemic burden, after adjustment of possible confounders in multivariable linear regression analysis (Table 4).

**Table 3:** Covariables of myocardial ischemia identified in logistic regression analyses.

Univariable logistic regression			
	OR	95% CI	p
Total plaque volume / 10 mm^3^	1.02	1.00–1.03	0.052
Non-calcified plaque volume / 10 mm^3^	1.02	1.00–1.03	0.052
Calcified plaque volume / 10 mm^3^	1.01	0.96–1.07	0.662
Positive remodelling	1.37	0.66–2.86	0.404
Age (years)	1.02	0.98–1.06	0.353
Female sex	0.77	0.38–1.56	0.465
Hypertension	1.99	0.88–4.50	0.100
Diabetes	0.81	0.29–2.26	0.682
Total cholesterol (mmol/l)	1.04	0.79–1.38	0.778
Statin treatment	1.26	0.60–2.66	0.543
Current smoker	0.68	0.25–1.86	0.447
Body mass index (kg/m^2^)	0.95	0.88–1.03	0.247
Obesity	0.37	0.14–0.94	0.036
Left ventricular mass index (g/m^2.7^)	1.06	1.02–1.11	0.006
Left ventricular ejection fraction (%)	1.07	1.07–1.13	0.029
Calcium score (per 10 units increase)	1.01	0.97–1.04	0.682
Segment involvement score	1.17	0.93–1.48	0.182

Multivariable logistic regression			

Model 1	OR	95% CI	p

Total plaque volume / 10 mm^3^	1.02	1.00–1.04	0.045
Age (years)	0.99	0.94–1.04	0.617
Female sex	1.19	0.52–2.71	0.679
Hypertension	1.80	0.74–4.40	0.199
Diabetes	0.71	2.35–2.12	0.533
Left ventricular mass index (g/m^2.7^)	1.06	1.01–1.11	0.016
Calcium score (per 10 units increase)	0.99	0.96–1.04	0.924
Positive remodelling	1.07	0.47–2.45	0.872

Model 2	OR	95% CI	p

Non-calcified plaque volume / 10 mm^3^	1.02	1.00–1.04	0.055
Age (years)	0.99	0.94–1.04	0.667
Female sex	1.22	0.53–2.79	0.637
Hypertension	1.77	0.72–4.33	0.211
Diabetes	0.72	0.24–2.16	0.558
Left ventricular mass index (g/m^2.7^)	1.06	1.01–1.12	0.017
Calcium score (per 10 units increase)	1.00	0.97–1.05	0.832
Positive remodelling	1.06	0.46–2.41	0.898

**Table 4:** Determinants of extent of myocardial ischemia found in linear regression analysis.

	Univariable		Multivariable (R^2^ = 0.17, p = 0.011)	
	β	p	β	p
Total plaque volume / 10 mm^3^	0.17	0.053	0.20	0.048
Non-calcified plaque volume / 10 mm^3^	0.16	0.082	N/A	N/A
Age (years)	0.003	0.975	−0.13	0.219
Female sex	−0.13	0.140	−0.05	0.579
Hypertension	0.20	0.024	0.21	0.025
Diabetes	0.06	0.510	0.02	0.847
Left ventricular mass index (g/m^2.7^)	0.30	0.001	0.25	0.009
Positive remodelling	0.03	0.743	−0.03	0.751
Calcium score (per 10 units increase)	0.08	0.402		
Statin treatment	0.11	0.228		
Current smoker	−0.08	0.397		
Body mass index (kg/m^2^)	−0.09	0.346		
Segment involvement score	0.11	0.219		
Calcified plaque volume / 10 mm^3^	0.10	0.277		

N/A, not applicable.

## 4 Discussion

The present study demonstrates that the total atherosclerotic plaque burden, assessed as total plaque volume by quantitative CCTA, was independently associated with presence of myocardial ischemia in patients with chronic coronary syndrome and non-obstructive CAD. Our findings expand current knowledge by demonstrating that larger total coronary plaque burden estimated by CCTA increases the risk of having myocardial ischemia in non-obstructive CAD independent of cardiovascular risk factors.

### 4.1 Myocardial ischemia in non-obstructive coronary artery disease

Myocardial ischemia was found in 53% of the participants despite having < 50% stenosis by CCTA. There are several possible mechanisms that may explain this finding. First, as the presence of at least one cardiovascular risk factor was an inclusion criterion, this is likely to underlie the high prevalence of myocardial ischemia found in our study. Second, a similar prevalence has been found in previous studies [27]. In fact, it was recently demonstrated that among patients with non-obstructive CAD by coronary angiography, near half of the patients had abnormal fractional flow reserve (FFR), reflecting an intracoronary pressure drop that is likely to cause myocardial ischemia [27]. Their results show that also non-obstructive CAD may impact coronary blood flow. Third, increased myocardial oxygen demand may contribute to myocardial ischemia in patients with non-obstructive CAD. In line with this, we previously demonstrated that left ventricular hypertrophy was associated with presence of myocardial ischemia in patients with non-obstructive CAD [12]. Forth, several pathophysiological mechanisms, including arterial stiffness, microvascular and endothelial dysfunction, are likely to contribute to myocardial ischemia in non-obstructive CAD [13], [28], [29]. For instance, endothelial dysfunction has been associated with presence of lipid rich plaques in patients with chest pain and non-obstructive CAD [29]. Whether the association between the total plaque burden and myocardial ischemia is independent of these mechanisms requires further investigation. The unexpected inverse association between obesity and myocardial ischemia in univariable analysis maybe due to a selection bias induced by the combination of reduced image quality by CCTA and an increased cardiovascular risk in obesity that favours direct referral to invasive coronary angiography in this subgroup of patients. In addition, the statistically significant higher left ventricular ejection fraction among patients with myocardial ischemia maybe due to an increased prevalence of left ventricular hypertrophy commonly associated with an increased left ventricular ejection fraction [30]. To reduce the risk of selection bias and collinearity, we therefore avoided to include obesity and left ventricular ejection fraction in the multivariable regression analyses. Finally, evidence of ischemia in patients with non-obstructive CAD has in the past been labelled as “false-positive”, in spite of several studies demonstrating that these patients have an impaired prognosis [3]. Our findings emphasize that the term “false-positive ischemia” should no longer be used for these patients. Our results underscore that the underlying mechanisms causing INOCA are likely multifactorial and should be further investigated in the individual patient in order to optimize patient outcome.

### 4.2 Myocardial ischemia and coronary artery plaque burden

We have previously demonstrated that the angiographic plaque burden was associated with myocardial ischemia independent of stenosis severity and cardiovascular risk factors in patients with non-ST elevation myocardial infarction [31]. In addition, a recent *meta*-analysis including 2123 patients, mostly with suspected CAD, indicated a quantitative relationship between the total atherosclerotic burden measured by coronary artery calcium score and stress-induced myocardial ischemia [8]. Of note, many of these studies did not report stenosis obstruction severity, so the relationship between calcium score as a marker of the total atherosclerotic burden and myocardial ischemia in non-obstructive CAD still remains unknown [8]. Few studies have reported the association between the total atherosclerotic plaque burden, including both calcified and non-calcified plaque, and ischemia in non-obstructive CAD [11]. In a sub-study from the CORE320 study, the total atherosclerotic burden was higher in patients with non-obstructive CAD who had myocardial ischemia than in those with normal perfusion [11]. However, there are some important aspects that should be kept in mind while comparing our results to the findings from the CORE320 study. First, the cardiovascular risk profile is higher in the CORE320 population due to study design. In the CORE320 study, it was an inclusion criterion to have clinically indicated coronary angiography within 60 days. Accordingly, it is reasonable that the 31 INOCA patients in the CORE320 trial had a higher cardiovascular risk than our population, where none of the participants had clinically indicated angiography. For instance, the prevalence of diabetes and current smoking was near two-fold higher in the INOCA patients in the CORE320 trial compared to our study population, although prevalence of female sex, hypertension and age were similar [11]. In fact, none of the patients in our study proceeded to coronary angiography after CCTA. Second, the calcium score was numerically lower in the patients with INOCA in the CORE320 report compared to our results. However, different CT scanners and software were used for calcium scoring, which has been shown to result in substantial differences [32]. Accordingly, it is difficult to establish the underlying reasons and clinical implications of the different calcium scores found in our study and in the patients with INOCA in the CORE320 report [11]. Finally, their report did not include multivariable analysis. Thus, the present results expand current knowledge by demonstrating that in symptomatic patients with chronic coronary syndrome and non-obstructive CAD, the total plaque burden was independently associated with myocardial ischemia.

In our study, the independent association of coronary plaque burden with myocardial ischemia was found when combining calcified and non-calcified plaque volumes in multivariable analysis. Previous studies suggest that non-calcified plaque estimated by CCTA is more likely to cause myocardial ischemia than calcified plaque [9], [33], [34]. Diaz-Zamudio *et al.* showed that the low-density, non-calcified plaque burden was associated with myocardial ischemia independent of stenosis severity [33]. However, these studies did not include the total plaque burden in multivariable analyses due to collinearity with the non-calcified plaque, limiting direct comparison with our findings [9], [33], [34]. As the main component of the total plaque burden was non-calcified in our study, our results are in line with these previous results, although the association between the non-calcified plaque burden and myocardial ischemia became attenuated in multivariable analysis.

Nonetheless, our findings expand these previous results by demonstrating that plaque quantification may identify patients with INOCA. A recent sub-analysis from the SCOT-HEART (Scottish computed tomography of the heart) trial demonstrated that the burden of low-density, non-calcified plaque quantified by CCTA, predicted myocardial infarction independent of calcium score [35]. Prognostic studies are needed to establish whether plaque quantification is useful for risk stratification in INOCA.

### 4.3 Clinical implications

Our results have some important clinical implications. The current study demonstrates that patients with greater burden of non-obstructive CAD have increased risk of having myocardial ischemia. Our findings underline that non-obstructive atherosclerosis may contribute to myocardial ischemia. However, the usefulness of coronary artery plaque quantification in clinical decision-making needs further exploration.

## 5 Study limitations

The present study has some limitations. The cross-sectional design is unsuited to identify any prognostic implication or causal associations between the total plaque volume and myocardial ischemia. The study was approved by the Regional Ethics committee as a cross-sectional study. Accordingly, clinical follow-up data is unfortunately not available. Further, because all patients had clinically indicated CCTA, a referral bias cannot be excluded. Due to study design, the prevalence of cardiovascular risk factors in our study was high, and generalizability to more healthy populations should be made with caution. The time from CCTA to assessment of myocardial ischemia could theoretically affected our results, but all patients had stable symptoms in the time between CCTA and assessment of myocardial ischemia. At the time the study was performed, there was a lack of recommendations for medical treatment of patients with non-obstructive CAD in the current guidelines. Accordingly, change in medical treatment after CCTA was not part of the study protocol and was done according to the treating physician’s discretion. Any change in medical therapy between CCTA and echocardiography could theoretically affect our results. The reported sensitivity and specificity for myocardial contrast stress echo for the detection of CAD is 88% and 77%, respectively, which is comparable to the diagnostic performance of radionuclide perfusion imaging and cardiac magnetic resonance perfusion imaging [25]. Myocardial ischemia was prevalent also in the patients with lower than age-predicted heart rate at peak stress. Of note, when assessing myocardial ischemia by myocardial contrast echocardiography, there is less of a need to achieve age predicted peak heart rate than for detection of wall motion abnormalities [36]. Therefore, we consider the probability of false negative ischemia assessment due to insufficient heart rate at peak stress to be low. Although plaque quantification by CCTA has been validated against intravascular ultrasound, this was not performed in the current study. The use of CT-FFR was not available at our institution.

## 6 Conclusion

In conclusion, total coronary artery plaque burden quantified by CCTA was independently associated with presence of myocardial ischemia in patients with symptomatic chronic coronary syndrome and non-obstructive CAD. Further studies are needed to evaluate whether plaque quantification is useful for clinical management of patients with non-obstructive CAD.

## Funding

Financial support was obtained from the MedViz Consortium, a collaboration between the University of Bergen, Haukeland University Hospital and Christian Michelsen Research, all Bergen, Norway, and the Western Norwegian Regional Health Authorities. None of the sponsors had any involvement in study design, data collection, analysis or interpretation of data, writing of the report, or in the decision to submit the paper for publication.

## Declaration of Competing Interest

The authors report no relationships that could be construed as a conflict of interest.
